# Multi-compartmental risk assessment of heavy metal contamination in soil, plants, and wastewater: A model from Industrial Gazipur, Bangladesh

**DOI:** 10.1007/s10661-025-13818-9

**Published:** 2025-03-15

**Authors:** Md. Sahariar Sahen, Md. Azizul Haque Khan Naim, Md. Sabbir Hosen, Md. Assaduzzaman Pranta, Mehedi Hasan, Md. Mostafizur Rahman, Shoeb Rahman, Aakash Welgamage Don

**Affiliations:** 1 Laboratory of Environmental Health and Ecotoxicology, Department of Environmental Sciences, Jahangirnagar University, Dhaka, 1342 Bangladesh; 2 Department of Leather Engineering, Khulna University of Engineering and Technology, Khulna, 9203 Bangladesh; 3 Biorefining Research Institute (BRI), Lakehead University, Thunder Bay, ON P7B 5E1 Canada; 4 Department of Chemistry and Biochemistry, Georgia Southern University, Statesboro, GA 30458 USA; 5 School of Pharmacy, Applied Sciences and Public Health, Robert Gordon University, Aberdeen, AB10 7GJ UK

**Keywords:** Bioaccumulation, Public Health Risk, Transfer Factor, Heavy Metal Pollution, Ecological Risk, Environmental Monitoring

## Abstract

Heavy metal contamination in industrial-agricultural regions poses global challenges, yet comprehensive risk assessment models addressing both ecological and human health impacts are scarce. This study introduces a novel multi-compartmental risk assessment framework applied to the Saldha River region of Gazipur, Bangladesh, a rapidly industrialising area experiencing significant environmental stress. Here, we analysed eight heavy metals (Cr, Pb, Cu, Fe, Mn, Zn, Ni, and Cd) in soil, wastewater, and plant samples (spinach, wild rice, and nut grass) via atomic absorption spectrophotometry (AAS). Ecological risks were evaluated through contamination factor (CF), pollution load index (PLI), and geo-accumulation index (I_geo_), while human health risks were assessed using hazard indices (HI). Results revealed severe Cd contamination (enrichment factor 2563.19), indicating substantial anthropogenic influence. Correlation analysis of wastewater samples showed strong associations between metal pairs, such as Cu–Zn (0.92), Cu-Fe (0.90) and Zn-Mn (0.87), indicating common industrial sources. Transfer factor (TF) analysis in plants demonstrated substantial variability in metal uptake, with Mn and Ni showing the highest bioavailability, increasing risks to local food chains. Human health risk assessments indicated hazard indices (HI) exceeding safety thresholds for both adults and children, underscoring the urgent need for mitigation strategies. This study offers a novel, integrative framework for assessing multi-source contamination and provides critical baseline data for future environmental policy development. The model is adaptable to industrial regions worldwide, such as textile hubs in Southeast Asia or metal processing zones in Europe and North America, offering new insights into contamination pathways and risk management.

**Graphical Abstract:**

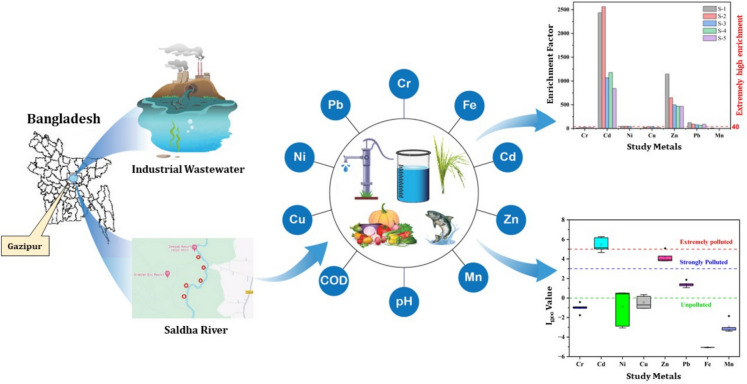

## Introduction

The widespread and emerging crisis of heavy metal contamination poses a significant threat to environmental and public health in both industrialised and developing nations worldwide. While heavy metals are naturally occurring elements, human activities have substantially intensified their prevalence in the environment (Ahmed et al., 2021). Industrial activities such as mining, smelting, and manufacturing processes that utilise metals like lead (Pb), iron (Fe), and cadmium (Cd) play a significant role in the release of these toxic elements into ecosystems (Adimalla et al., 2020; Rahman et al., 2019). For example, in 2019, the generation of electronics-related waste, which is predominantly metal-rich, reached 53.6 million metric tons globally (Frazzoli et al., 2022). Projections indicate that by 2030, this volume will increase to 74.7 million metric tons. Approximately 80% of this electronic waste is transferred to low- and middle-income countries, including India, Nigeria, Brazil, Ghana, and Pakistan, primarily due to the lower cost of disposal and weaker environmental regulations (Sthiannopkao & Wong, 2013), creating segregated contamination areas in South Asia, Africa, and South America.

The rising demand for metals is driven by rapid industrialisation, enhanced living standards, and the increasing prevalence of electronic devices, including electric vehicles. For instance, as electric vehicles become more widespread, the requirement for recycling or disposing of their batteries will grow substantially (Raabe, 2023). This trend indicates that the continued transport of metal waste to certain global regions will result in the creation of heavy metal-contaminated hotspots around the world. It must be taken into consideration that despite the localised nature of these contamination sites, the interconnectedness of food webs implies that such pollution can indirectly impact the entire world. Metals can migrate through plant uptake, aquatic organisms, and water systems (Vincent et al., 2022), resulting in their nondeliberate reintroduction into our food supply across geographical boundaries, posing a persistent threat to public health.

Numerous studies indicate that heavy metals from industrial and agricultural sources infiltrate aquatic and groundwater systems and pose considerable risks to ecosystems and public health due to their toxicity and bioaccumulation (Rakib et al., 2022). Currently, surface waters (e.g., rivers, lakes) and groundwater in many parts of the world are heavily impacted by the discharge of untreated industrial waste, predominantly containing metals such as Pb, Fe, Cd, chromium (Cr), manganese (Mn), and copper (Cu) (Jehan et al., 2020; Proshad et al., 2021; Vincent et al., 2022). For example, textile industries discharge considerable amounts of Cr, Cu and zinc (Zn) into waterways during dyeing and finishing processes (Velusamy et al., 2022). These metals contaminate the environment, bioaccumulate in aquatic life, and ultimately enter the human food chain, causing health issues like neurotoxicity and cancer (Kumar et al., 2017; Islam et al., 2018).

Contamination of water and irrigation systems by heavy metals leads to the uptake of these toxins by food crops. Studies have demonstrated that rice irrigated with water high in arsenic (As) accumulates substantial amounts of this toxin, significantly endangering the health of populations in countries like Bangladesh, Sri Lanka, India, and China, where rice is a dietary staple (Proshad et al., 2018). Similarly, spinach and other leafy vegetables like lettuce and kale, grown in Cd-contaminated soil, can absorb high concentrations of this metal, and their regular consumption can lead to severe health issues, such as kidney damage and increased bone fragility (Hossain et al., 2021). Therefore, understanding the level of contamination, its sources, and the potential health impacts is crucial to safeguard public health and ensure food safety.

Agriculture is a major economic sector in the district, contributing approximately 57.46% of the local GDP (Manik, 2023). Despite its importance, intensive agricultural practices, such as using fertilisers, pesticides, and extensive land manipulation, pose significant risks for heavy metal contamination. These practices can lead to the leaching, diffusion, infiltration, and accumulation of heavy metals from fertilisers, insecticides, and pesticides into river sediments and biota. Consequently, these metals can bioaccumulate in crops and aquatic organisms, entering the human food chain and posing health risks. Once metals enter a river system, they distribute between water and sediments, affecting water quality and stability and acting as reservoirs that pose a significant threat to aquatic life. (Hossain et al., 2021; Rakib et al., 2021).

Our study addresses potential gaps in understanding the extent and impact of heavy metal contamination in water, soil, and commonly found plant materials in regions influenced by industrial activities along the Shaldha River. Despite known risks, detailed regional data on the spread of contamination and its direct impact on public health and the environment remain scarce. This study investigates the dispersion of hazardous metals such as Pb and Cr into food since these metals are linked to severe health issues, including neurological deficits and lung cancer (Rehman et al., 2018). Thus, the primary objectives of this study are to quantify the concentrations of commonly occurring heavy metals (Cr, Ni, Cd, Cu, Pb, Fe, Zn, and Mn) in soil, wastewater, and plant samples and to assess their environmental impact through ecological and pollution indices such as the geo-accumulation index (I_geo_), enrichment factor (EF), contamination factor (CF), and pollution load index (PLI). By integrating data from multiple environmental compartments, this study provides a novel and comprehensive risk assessment model that can be adapted to other industrial regions worldwide, offering valuable insights for global environmental policies. The findings of this study also contribute to the development of targeted mitigation strategies that can help local authorities address the growing threat of heavy metal contamination in Bangladesh and beyond.

## Methodology and materials

### Study area

Gazipur is a major industrial city located in the central region of Bangladesh, bordered by Mymensingh and Kishoreganj to the north, Narshingdi to the east, Tangail to the west, and Dhaka and Narayanganj to the south. Over the past few decades, Gazipur has emerged as one of the most significant industrial hubs in Bangladesh, alongside Dhaka, Khulna, and Chattogram. However, this industrial expansion has often been accompanied by lax enforcement of environmental regulations, leading to substantial ecological degradation (Hossain et al., 2020).

The rapid urbanisation and industrialisation of Gazipur have dramatically transformed its land-use patterns. A study by Arifeen et al. (2021) on land-use and land-cover (LULC) changes between 1990 and 2020 revealed considerable urban expansion which is associated with a marked decline in agricultural land in Gazipur. Gazipur now hosts over 1,773 industrial factories, encompassing a diverse range of industries, including textiles, apparel, footwear, paper mills, paints, ceramics, and packaging (Arifeen et al., 2021; Bangladesh Bureau of Statistics, 2020; Jiku et al., 2021). These industries are key contributors to both regional and national economic growth (Abdullah et al., 2019; Hossain et al., 2019).

**Fig. 1:**
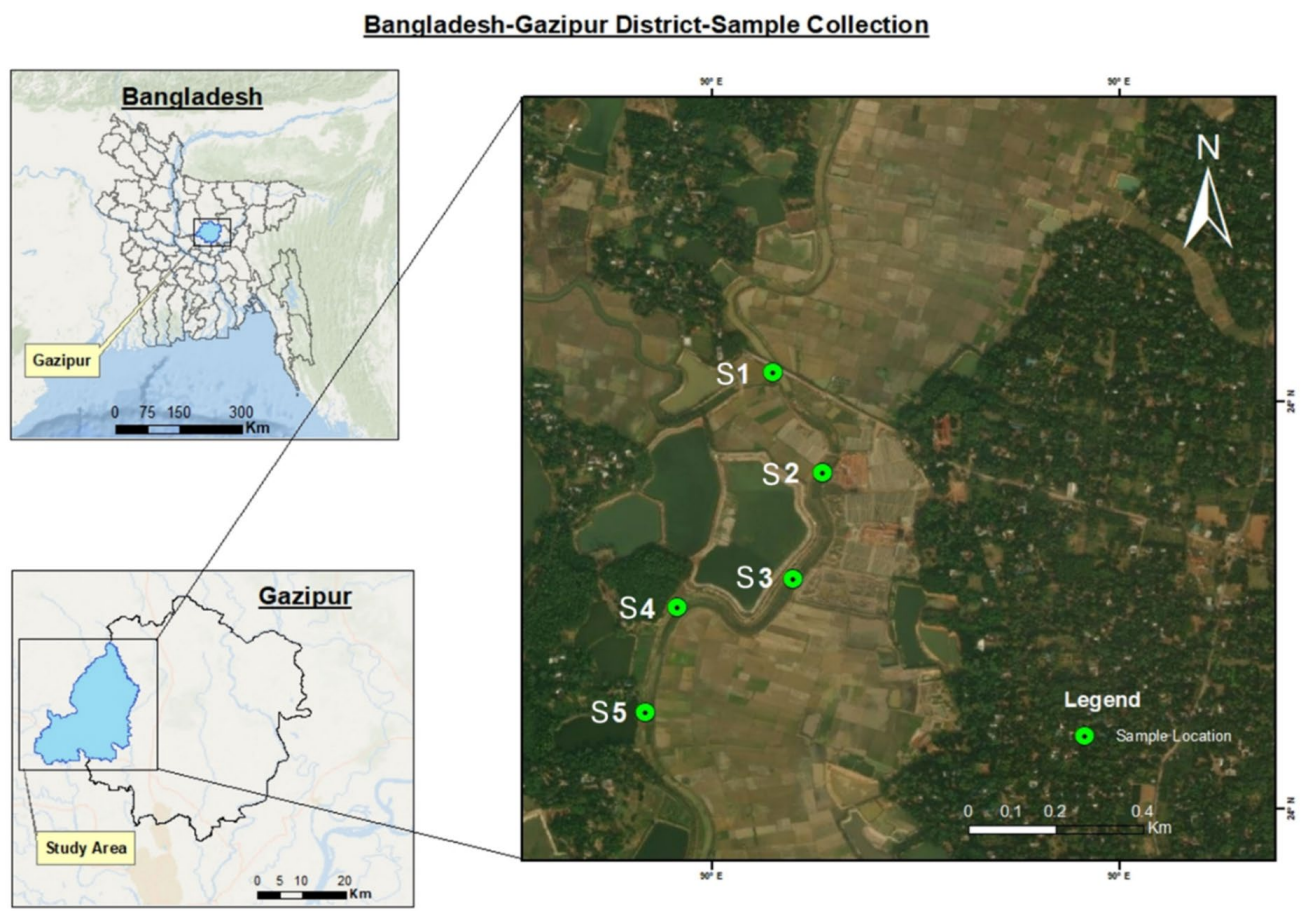
Locations (S1-S5) of the sampling points in the study area of Gazipur District, Dhaka, Bangladesh (Map data ©2024 Google)

This study was conducted in Monipur, Hotapara, one of the most densely populated (1.8 million) and industrialised areas within Gazipur city (Fig. 1). The Shaldha River seen in Fig. 1 is a tributary of the ancient Bongshai River which flows west of the Mirzapur Union and meanders through these industrial zones in the city, making it particularly vulnerable to pollution due to its proximity to concentrated industrial activities. Local villagers heavily use the riverbanks for agriculture, creating a complex interaction between industrial pollution and local farming practices. In addition, most factories in the area lack adequate wastewater treatment facilities which leads to the discharging of untreated effluents directly into the river or onto agricultural land, posing significant environmental risks (Cheshmazar et al., 2018).

Gazipur features a tropical climate with distinct wet and dry seasons, receiving an average rainfall of 2,036 mm during the wet season and maintaining an average annual temperature of 25.8°C. The terrain is characterised by dissected terraces and valleys with predominantly nutrient-poor acidic clay soils (UNDP/FAO, 1988).

### Sample collection

The sampling sites (Fig. 1) were carefully selected to capture the extent and variability of heavy metal contamination in the study area, and also to ensure the relevance and applicability of the findings to similar contaminated environments. Five samples of soil, discharged wastewater, and plant material were collected from five distinct locations (Fig. 1). The selected locations were chosen based on their proximity to major industrial effluent discharge points into the Shaldha River which is a key area of concern due to its exposure to untreated industrial wastewater.

The first site (S1, located at 24°07′38.4"N, 90°22′32.0"E) represents the primary discharge point, where the majority of industrial effluents are released into the river through a combined pipeline. The other sites, S2 (24°07′32.8"N, 90°22′27.7"E), S3 (24°07′30.8"N, 90°22′25.2"E), S4 (24°07′30.9"N, 90°22′22.9"E), and S5 (24°07′28.6"N, 90°22′20.2"E), are located downstream and represent individual point sources where specific factories discharge effluents directly into the river via separate pipelines. These sites were selected to provide comprehensive spatial coverage of both the main pollution entry point and its downstream impacts. All samples were collected during the dry season, from December 2023 to February 2024, to avoid the weather-related challenges (e.g., excessive rain and sediments) posed by the monsoon season (Khan et al., 2023).

In each location, 20 g of agricultural soil was collected from a 10–15 cm depth using standardised procedures described elsewhere (Addis & Abebaw, 2017). Additionally, samples of newly grown grass *Enhydra fluctuens* were collected from the sampling position on the bank of the canal. Each soil and plant sample was securely stored in autoclaved glass jars to prevent contamination. The wastewater samples were collected in 250 ml HDPE bottles, chosen for their chemical inertness and suitability for containing liquids without leaching. Samples were transported to the laboratory in a cooler box with ice packs to ensure refrigeration during transit and prevent biochemical degradation or changes in sample composition.

### Sample preparation

The collected soil and grass samples (*E. fluctuens*) were sun-dried for 48 h to reduce their initial moisture content. They were then dried in an oven at 105 °C for 24 h to remove residual moisture. After oven drying, the samples were transferred to a desiccator and maintained at room temperature. Their weights were monitored and recorded at regular intervals until a consistent weight was achieved. Samples were then ground into a fine powder using a laboratory grinder to ensure homogeneity and were stored in clean, air-tight glass jars until acid digestion.

The soil and grass material were digested using EPA method 3050B, as previously described (Álvarez et al., 2021). Nitric acid was added to 2 g of each sample and was heated to 150 °C or until a brown fume emerged. For each sample, 50 mL of deionised water was added and heated to 70 °C for one hour. After allowing the suspensions to cool down to room temperature, they were filtered using filter paper (Whatman No. 1). The final volume of all samples was adjusted to 100 ml by adding distilled water.

To prepare the water samples for heavy metal analysis, 50 ml was taken from each sample bottle. Then, 2 ml of nitric acid was added, and the mixture was heated for 2 h at 120 °C. After heating, the samples were cooled for 15 min. The sample volume was then adjusted to 50 ml with distilled water, filtered (Whatman No. 1 filter paper) and refrigerated (4 °C) until analysis.

### Physicochemical parameters of samples

The collected water samples were subjected to analytical testing to determine parameters such as pH, electrical conductivity (EC), chemical oxygen demand (COD), dissolved oxygen (DO), and total dissolved solids (TDS). These analyses were conducted following the guidelines outlined by Manivasakam (2005) and the procedures specified by the American Public Health Association (American Public Health Association, 2012).

### Assessment of soil pollution

#### Geo-accumulation index (I_geo_)

The geo-accumulation index (I_geo_) provides information on the level of metal pollution in the soil. The below-mentioned formula, generated by Müller (1969) and widely utilised (Islam et al., 2020; Kumar et al., 2021), was used to compute the I_geo_.

1 $${I}_{geo} = {log}_{2} \left(\frac{{C}_{m}}{1.5{B}_{m}}\right)$$

In this analysis, C_m_ represents the concentration of metals detected in the soil samples, while B_m_​ denotes the background value of the same metals. To account for potential fluctuations in the background data, a factor of 1.5 was applied (Islam et al., 2020), enhancing the robustness of the comparison against baseline values. For the baseline, we utilised the published global average concentrations of metals in shale as previously reported (Shaw et al., 1976; Wedepohl, 1995). The geo-accumulation index calculated from these values categorises pollution into seven distinct classes, each representing a different level of pollution severity, as seen in Table 1. This classification helps systematically evaluate the extent of metal accumulation and its environmental impact.

**Table 1:** Seven classes of the geo-accumulation index (Müller, 1969)

Class	I_geo_ Values	Contamination level
0	I_geo_ < 0	almost uncontaminated
1	0 < I_geo_ < 1	uncontaminated to rather polluted
2	1 < I_geo_ < 2	somewhat contaminated
3	2 < I_geo_ < 3	moderately to deeply contaminated
4	3 < I_geo_ < 4	deeply contaminated
5	4 < I_geo_ < 5	strongly to extremely contaminated
6	5 < I_geo_	extremely contaminated

#### Enrichment factor (EF)

Since metals can originate from natural and artificial sources, a normalised enrichment factor is frequently utilised to differentiate between the origins of metals in soils (Islam et al., 2018; Pandey et al., 2016). The normalisation of soil metal content against a trace reference metal (e.g., Al, Fe, Mn, Ti, Sc, Li, and Cs) is known as the EF (Karbassi et al., 2008; Salati & Moore, 2010). In this study, Fe was chosen as the reference metal due to its increasing prevalence in EF calculations (Bhuiyan et al., 2010; Kumar et al., 2021). Additionally, Fe demonstrated the most stable concentrations across the study area compared to other potential reference trace metals. The EF was calculated using the following formula:

2 $$\text{EF }= [({\text{C}}_{\text{m}}/{\text{Fe}}_{\text{s}}{)}_{\text{sample}}] / [({\text{C}}_{\text{m}}/{\text{Fe}}_{\text{s}}{)}_{\text{shale}}]$$

In this study, the (C_m_/Fe_s_)_sample_ represents the ratio of the metal concentration in soil (C_m_) to the concentration of iron (Fe_s_) in the soil sample. Similarly, (C_m_/Fe_s_)_shale_ is the same ratio in shale.

Based on EF values, five distinct categories of contamination have been identified as described in Table 2 (Küçüksümbül et al., 2022):

**Table 2:** Distinct categories of enrichment factor

EF	Contamination
EF < 2	No or minimal
2 ≤ EF < 5	Moderately
5 ≤ EF ≤ 20	Significantly
20 ≤ EF < 40	Very Strongly
EF > 40	Extremely

#### Contamination factor (CF) and pollution load index (PLI)

The CF is the ratio of the metal concentration in a soil sample (C_m_) to its corresponding background concentration (B_m_) in shale. It is calculated as follows:

3 $$\text{CF }= {\text{C}}_{\text{m}} / {\text{B}}_{\text{m}}$$

Based on CF values, contamination can be classified into the following categories, as seen in Table 3 (Jolly et al., 2023).

**Table 3:** Distinct categories of contamination factor

CF	Contamination
CF < 1	No or minimal
3 ≤ CF ≤ 6	Significantly considerable
CF > 6	Very Strongly

The Pollution Load Index (PLI) provides an overall assessment of the toxicity status of soil samples. It is calculated as the n^th^ root of the product of the CF of each sample for the examined constituents (Tomlinson et al., 1980). The PLI was calculated using the below-mentioned equation (Tomlinson et al., 1980):

4 $$\text{PLI }= ({\text{CF}}_{1} \times {\text{CF}}_{2} \times {\text{CF}}_{3} \times {\text{CF}}_{4} \times {\text{CF}}_{5} \times \dots \dots \dots \dots . \times {\text{CF}}_{\text{n}}{)}^{1/\text{n}}$$

CF represents the contamination factor of each metal, and n represents the total number of metals analysed in the sample. A PLI value below 1 indicates no pollution load, while a value equal to or greater than 1 suggests the presence of pollution.

#### Potential ecological risk index (PERI)

The Potential Ecological Risk Index (PERI) is a widely used tool in contamination research to quantify the risk of metal deposition in soil to human health and ecosystems (Ciupa et al., 2020; Kara, 2020). The PERI is calculated using the following formulas (Hakanson, 1980);

5 $${\text{c}}_{\text{f}}^{\text{i}} = \frac{{\text{C}}_{\text{d}}^{\text{i}}}{{C}_{r}^{i}}$$

6 $$\text{ER }= {\text{E}}_{\text{r}}^{\text{i}} = {\text{T}}_{\text{r}}^{\text{i}} \times {\text{C}}_{\text{f}}^{\text{i}}$$

7 $$\text{PERI }= \sum_{i=1}^{n}{E}_{\text{r}}^{\text{i}}$$

${\text{C}}_{\text{d}}^{\text{i}}$ represents the metal quantity in soil, while, ${C}_{r}^{i}$ is the background value of the metal, ${\text{C}}_{\text{f}}^{\text{i}}$ is the contamination coefficient, ${\text{T}}_{r}^{\text{i}}$ is the biological toxic response factor and ${\text{E}}_{r}^{\text{i}}$ represents the ecological factor for each metal. Toxic response factors for Cr, Cd, Ni, Cu, Zn, and Pb are used as 2, 30, 5, 5, 1, and 5, respectively (Hakanson, 1980; Jolly et al., 2023). The following table (Table 4) presents the classification of ER and PERI categories based on established literature (Küçüksümbül et al., 2022):

**Table 4:** Ecological risk levels

ER	PERI	Level of ecological risk
ER < 40	PERI < 150	Low
40 ≤ ER < 80	150 ≤ PERI < 300	Moderate
80 ≤ ER < 160	300 ≤ PERI < 600	Considerable
160 ≤ ER < 320	**-**	High
ER ≥ 320	PERI ≥ 600	Very high

#### Soil-vegetable transfer coefficient / transfer factor (TF)

The TF is a metric used to quantify the changes in the bioavailability of metals to plants. According to the methodology outlined by Kachenko and Singh (2006), the TF was calculated as the ratio of the concentration of a metal in vegetables to its concentration in the surrounding soil. A higher TF indicates more efficient absorption of metals by the plants or less effective retention by the soil. Conversely, a lower TF suggests strong adsorption of metals to soil colloids (Habte et al., 2023).

#### Human exposure assessment

##### Non-carcinogenic risk assessment

The accumulation of heavy metals in the food chain poses a significant risk to human health. Various metals have been linked to adverse effects on the brain, while some are associated with cancer (Habib et al., 2024). To evaluate the potential short- and long-term health risks associated with heavy metal exposure from vegetable and water consumption among the Hotapara population in Gazipur City, we calculated the estimated daily intake (EDI), chronic daily intake (CDI), hazard quotient (HQ), and cancer risk (CR) using guidelines from the US Environmental Protection Agency (USEPA, 1989, 1997, 2001, 2002). The parameter values used in these calculations are detailed in Table 5. The oral reference dose (RfD) value is the oral reference dose that was determined following Küçüksümbül et al. (2022). Typically, HQ < 1 indicates no adverse health effects, while an HQ > 1 signifies the likelihood of adverse health effects (Enuneku et al., 2018). The HQ for each element was determined using the following equations.

**Table 5:** Input parameters to characterise the CDI and CR value

Parameters	Description	Unit	Adult	Children
CS	Concentration of metal	mgkg^−1^	-	-
IngR	Ingestion rate per unit of time	mday^−1^	100	200
EF	Exposure frequency	daysyear^−1^	350	350
ED	Exposure duration	Years	30	6
BW	Body weight	Kg	70	15
AT	Average time	Days	365*70	365*70
CF	Conversion factor	Kgmg^−1^	10^–6^	10^–6^
AF	Adherence factor	mgcm^−2^	0.07	0.2
SA	Exposure skin area	cm^2^	5700	1600
ABS_d_	Dermal absorption fraction	-	0.01	0.001
InhR	Inhalation Rate	m^3^day^−1^	20	20
CSF	Chronic oral slope factor	mgkg^−1^day^−1^	Pb = 0.0085, Cr = 4.1, Cd = 6.3, Ni = 0.84	
ABS_GI_	Gastrointestinal absorption factor	-	Pb = 1, Cr = 0.013, Cd = 0.025, Ni = 0.04	
IUR	Chronic inhalation unit risk	(µg m^−3^)^−1^	Pb = 0.000012, Cr(iii) = 0.0012, Cd = 0.0018 Ni = 0.00026	
PEF	Particle emission factor	m^3^ kg^−1^	1.36 × 10^9^	
ET	Exposure Time	h d^−1^	24	

8 $${CDI}_{ing}= \frac{CS \times IngR \times EF \times ED }{BW \times AT}\times CF$$

9 $${CDI}_{dermal}= \frac{CS \times SA \times AF\times {ABS}_{d} \times EF \times ED }{BW \times AT} \times CF$$

10 $${CDI}_{inh}= \frac{CS \times InhR \times EF \times ED \times EF }{BW \times AT} \times CF$$

11 $$HQ = \frac{CDI}{RfD}$$

12 $$\text{HI }= \sum \text{ HQ }=\text{ HQing }+\text{ HQdermal }+\text{ HQinh}$$

##### Carcinogenic risk assessment

The likelihood that a person will get cancer of any kind as a result of being exposed to dangerous substances like Pb over their lifetime is known as their carcinogenic risk. Regarding carcinogenic risk, values less than 10^–6^ are deemed acceptable, ranges from 10^–6^ to 10^–4^ are considered tolerable, and values higher than 10^–4^ are considered harmful (Lim et al., 2008). The following equations were used to determine the carcinogenic risk (USEPA, 1989, 1997, 2001, 2002):

13 $${CR}_{ing}= \frac{CS \times AF \times IngR \times EF \times ED \times CF \times {CSF}_{ing}}{BW \times AT}$$

14 $${CR}_{dermal}= \frac{\text{CS }\times \text{ SA }\times \text{ AF }\times \text{ ABSd }\times \text{ EF }\times \text{ ED }\times \text{ CF }\times \text{ CSFd }\times {\text{ABS}}_{\text{GI}}}{\text{BW }\times \text{ AT}}$$

15 $${CR}_{inh }= \frac{\text{CS }\times \text{ ET }\times \text{ EF }\times \text{ ED }\times \text{ IUR }\times {10}^{3}}{\text{PEF }\times 24 \times \text{ AT}}$$

16 $$\text{Total carcinogenic risk CRtotal}=\sum \text{Risk}=\left({\text{CR}}_{\text{ing}}+{\text{CR}}_{\text{dermal}}+{\text{CR}}_{\text{inh}}\right)$$

### Statistical analysis

The data were summarised using the mean value, standard deviation and range of the analysed samples. The value represented in the box plot summarising data distribution includes the 25% ~ 75% quartile, mean and median. A statistical analysis was conducted using STATA 13.0 (StataCorp LLC) and plotted by OriginPro 2024 (OriginLab Corporation). Correlation analyses were performed by stepwise selection with a significance level of *p* < 0.05.

Pearson's correlation coefficient (r) is a statistical measure that quantifies the strength and direction of a linear relationship between two variables. The correlation coefficient is calculated using the following equation:

17 $$r= \frac{\sum {(x}_{i }-\overline{x } ) {(y}_{i }-\overline{y })}{\sqrt{{\sum {(x}_{i }-\overline{x } )}^{2}{\sum {(y}_{i }-\overline{y } )}^{2}}}$$

In this context, X and Y represent the two variables being analysed, and n denotes the number of data points. The correlation coefficient, r, ranges from −1 to + 1. An r-value close to + 1 indicates a strong positive linear correlation, suggesting that as X increases, Y also tends to increase. Conversely, an r-value close to −1 indicates a strong negative linear correlation, meaning that as X increases, Y tends to decrease. When the r-value is 0, it suggests no linear relationship between the variables.

## Results and discussion

### Soil analysis

#### Spatial distribution of heavy metals in soil

The soil samples from five different sites along the Saldha River (Fig. 1) were tested for the presence of eight different metals using AAS, and their mean concentrations are documented in Table 6, compared with the permissible thresholds established by the European Standards 2010 and Food and Agricultural Organization (FAO, 2004). The concentrations of each metal, including Cr, Cd, Ni, Cu, Zn, Pb, Fe, and Mn, varied between 39 and 100.95, 11.39 and 35.1, 12.14 and 146.71, 32.54 and 85.54, 1983.63 and 4832.35, 62.19 and 109.52, 2096.71 and 2157.62 and 121.64 and 355.44 mg/kg respectively.

**Table 6:** Heavy metal content in the soil (mg/kg)

Heavy metals	Mean	Germany*	Netherlands*	Sweden*	FAO, 2004
Cr	69.55 (± 21.77)	60.0	30.0	60.0	100.0
Cd	21.90 (± 10.99)	1.0	0.5	0.4	1.0
Ni	90.76 (± 71.13)	50.0	15.0	30.0	50.0
Cu	53.58 (± 25.23)	40.0	40.0	40.0	100.0
Zn	2755.63 (± 1207.36)	150.0	100.0	100–150	200.0
Pb	80.90 (± 17.74)	70.0	40.0	40.0	50.0
Fe	2134.11(± 24.52)	-	-	-	-
Mn	183.99 (± 97.03)	-	-	-	-

*European Commission Director General Environment, ECDGE 2010

Zn and Cd were particularly prominent, with concentrations exceeding the highest permissible limits set by European standards by 32.22 and 54.75 times, and 13.78 and 21.9 times of highest limit set by FAO, respectively (Table 6). This suggests significant pollution from local industrial activities such as electroplating, mining, and agricultural runoff (Lone et al., 2008). It was noted that all of the metal concentrations recorded were above the permissible level set by Germany, Netherlands, and Sweden as well as the permissible limit set by FAO (Table 6).

As described above, Gazipur is a rapidly industrialising area in Dhaka, and the Shaldha River flows through this industrial zone and adjacent towns, making it particularly susceptible to heavy metal pollution. The collected soil samples likely accumulated these metals through various anthropogenic activities, primarily due to the leaching of metals from landfills, refuse dumps, excrement, animal and chicken manure, and industrial activities such as metal mining, smelting, and foundries. Numerous metal-based industries in the area could potentially contribute to soil contamination, as industrial discharges often contain high levels of heavy metals, which are prone to accumulate in the soil and water bodies.

#### Soil pollution assessment for metals

##### Geo-accumulation index (_Igeo_)

**Fig. 2:**
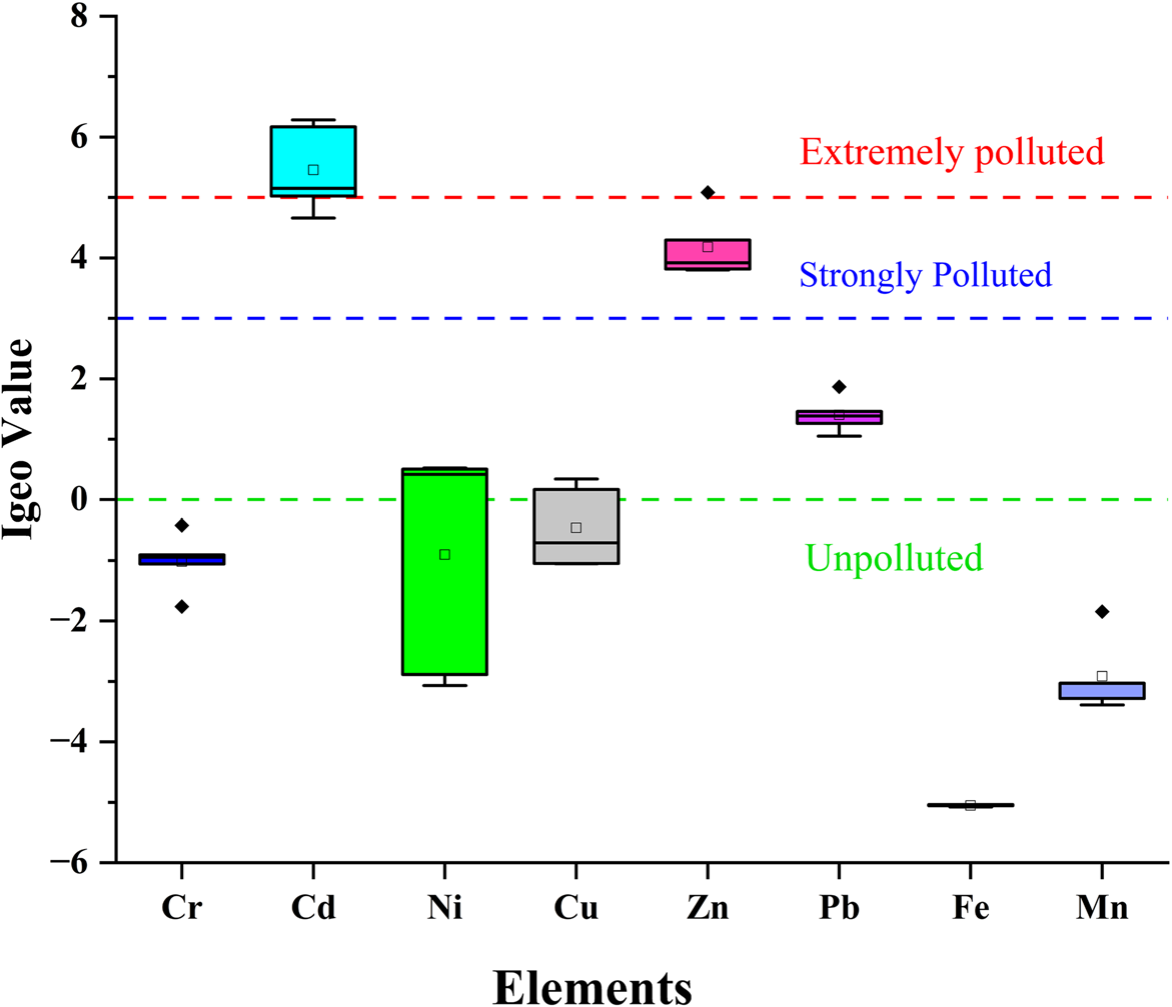
Geo-accumulation index (I_geo_) for heavy metals in soils in the study area

The aim of evaluating the I_geo_ in this study was to evaluate the levels of heavy metal contamination within the soil of the Shaldha River and to determine whether their origin is geogenic or anthropogenic. Using I_geo_, we measured the contamination levels of several heavy metals such as Pb, Mn, Fe, Cu, Ni, Cd, and Cr, as depicted in Fig. 2. This method allowed for quantifying the extent of pollution and identifying potential primary sources of metals in the sampling area. As seen in Fig. 2, there are considerable variations in the contamination levels among the studied metals. Amongst them, Cd and Zn were observed to have the highest level of contamination (Fig. 2), suggesting substantial anthropogenic contributions. Studied by Kachoueiyan et al. (2024) also revealed that, according to the geoaccumulation index (Igeo), the sediments in Shahid Rajaee Reservoir were highly polluted with Pb and Zn. The anthropogenic sources of Cd in groundwater and soil primarily include landfills, metal industries, mining activities, traffic emissions, and sewage sludge (Sidhu et al., 2017). Similarly, the use of phosphate fertilisers, which often contain Cd as an impurity, is a major contributor to elevated Cd levels in soil and water ecosystems (Jeong et al., 2020; Kubier & Pichler, 2019). It must be noted that Cd exposure (i.e., through contaminated water) can lead to various health issues, primarily affecting the kidneys and bones. Long-term exposure, even at low levels, can cause kidney damage, including decreased function and chronic kidney disease (Han et al., 2020). The I_geo_ values for Ni and Cu fell into the moderately polluted category. Thus, they could originate from a mix of natural and industrial sources. Trace amounts of Ni and Cu are naturally present in water due to the weathering and erosion of ore-bearing rocks in the Earth's crust (Jehan et al., 2020). However, elevated levels of Ni in environmental samples can be directly attributed to anthropogenic activities such as electroplating, automobile emissions, battery disposal, and electronic waste (Rashid et al., 2021). Similarly, anthropogenic contamination of water and soil by Cu could be due to the corrosion of plumbing materials, including copper pipes, fittings, and brass faucets (Ahmad et al. 2021). In contrast, the negative I_geo_ values reported for Cr, Fe, and Mn (Fig. 2) indicate that they likely originated from natural, geogenic sources such as soil and rock weathering, not anthropogenic activities (Jabeen et al., 2023).

##### Enrichment factor (EF)

**Fig. 3:**
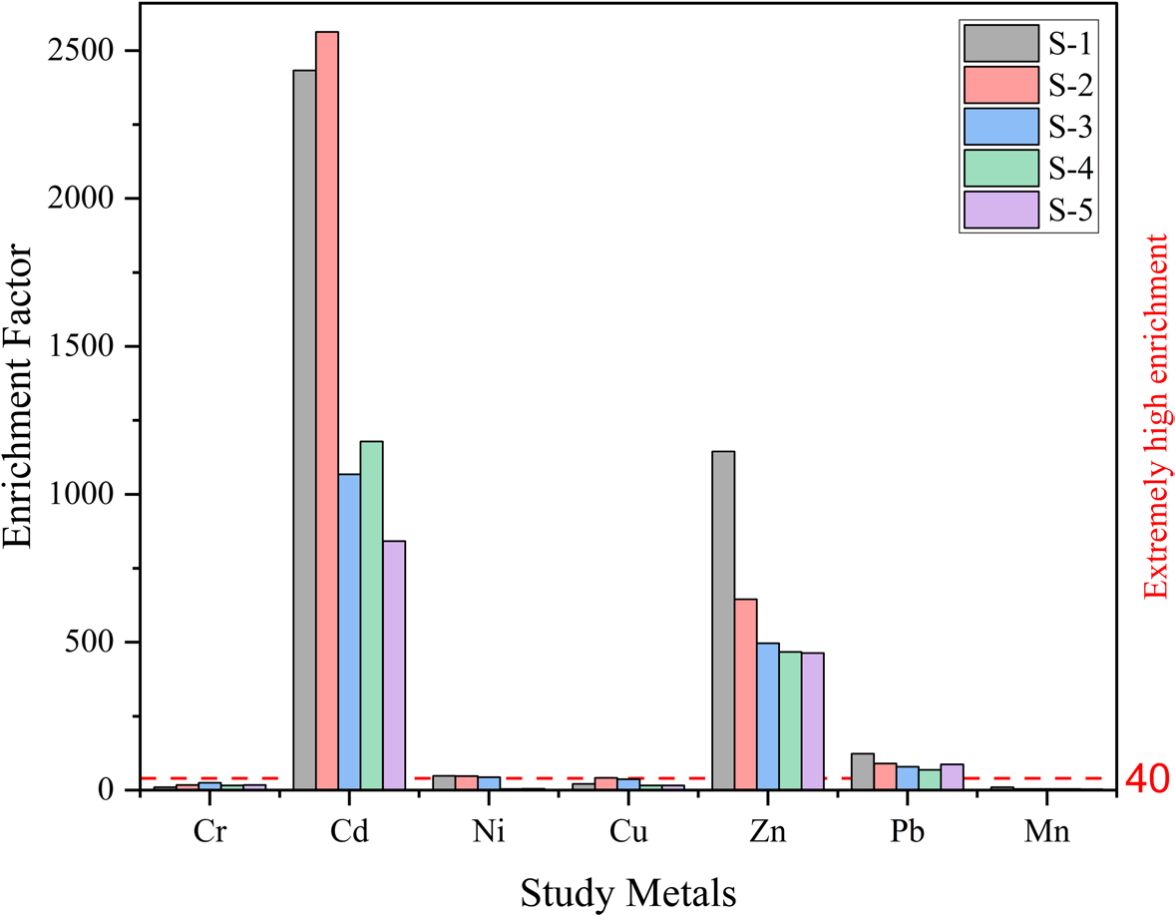
Enrichment factor for heavy metals in soils in the research area

The EF analysis was carried out to determine the extent of anthropogenic influence on heavy metal contamination in soil collected from various sampling sites along the Shaldha River. As illustrated in Fig. 3, the EF values for several metals exceed the threshold of 1.5, suggesting an anthropogenic influence on their elevated concentrations. The Cd shows the highest EF values across all sites, exceeding 2500 at sites S-1 and S-2, indicating severe contamination likely due to industrial discharges and agricultural runoff. This aligns with similar Cd pollution levels previously reported in some industrial areas of China and India (Sah, 2023; Si et al., 2019). The presence of diverse industries in the Shalda River region, including garment manufacturing, footwear production, plastics, ceramics, and paint factories, along with extensive agricultural activities (Ahmed et al., 2020), is a plausible contributor to the elevated Cd deposition observed in the soil. In addition, Zn and Pb also show notable enrichment, particularly at sites S-1 and S-2 for Zn and S-1 for Pb, suggesting substantial anthropogenic input. Although the EF for Cu and Ni were notably lower than those for Cd and Zn, they still reveal considerable human-induced contamination, particularly at sites S-2 and S-3 for Cu and S-1 for Ni. In contrast, the EF values for Cr and Mn were below the threshold, suggesting minimal anthropogenic impact and also indicating that their presence is mainly geogenic.

##### Contamination factor (CF) and pollution load index (PLI)

**Fig. 4:**
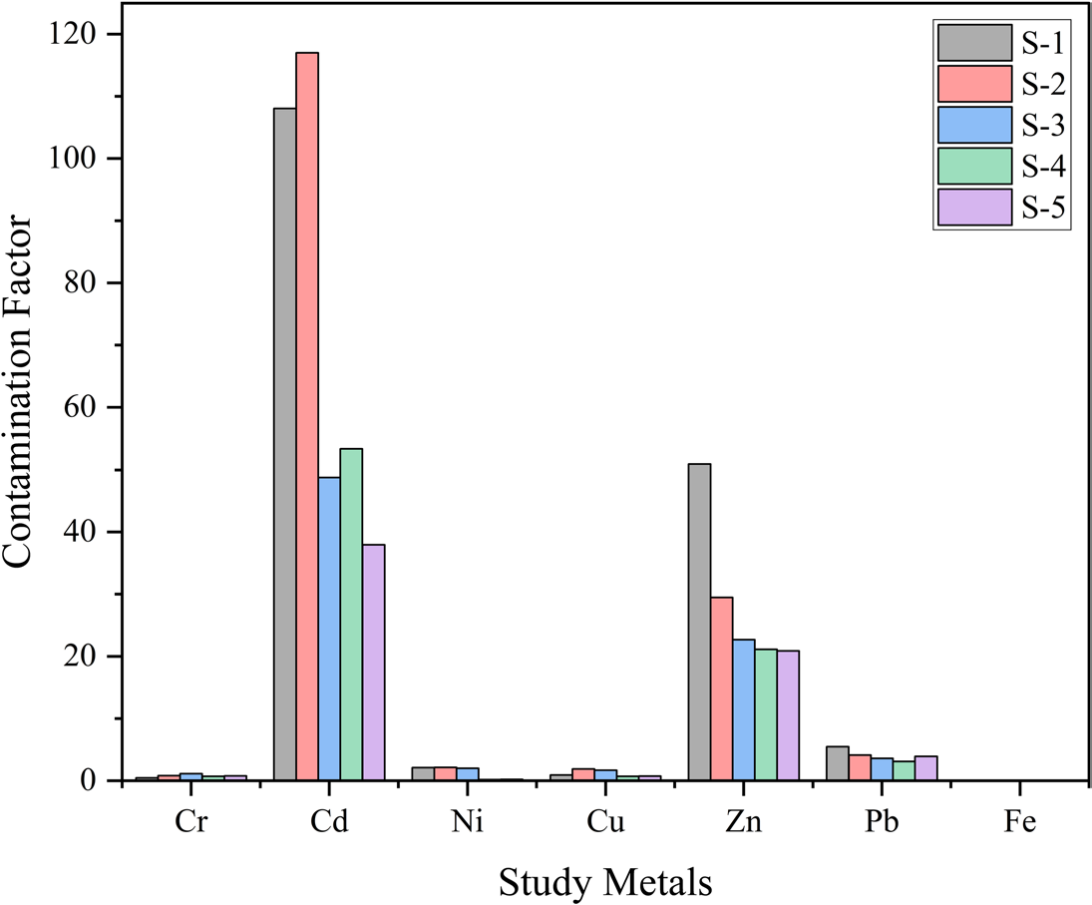
Contamination factor for the heavy metals in the research area

The CF is a valuable tool for assessing the degree of contamination resulting from specific anthropogenic activities (Jolly et al., 2023; Tomlinson et al., 1980). In our study, CF values for analysed metals ranged from 0.05 to 53.30 (Fig. 4), with Cd and Zn exhibiting the highest levels. This suggests a substantial anthropogenic influence on their elevated concentrations, potentially from the industrial activities and agricultural runoff in the surrounding area. Similar findings have been reported in Bangladesh, where industrial activities in the Dhaka region have led to significant Cd and Zn contamination in soils and water bodies (Majed et al., 2022). In India, Cd and Zn contamination has been linked to the extensive use of phosphate fertilisers and industrial effluents, leading to bioaccumulation in crops and subsequent health issues for consumers (Sharma et al., 2006). The elevated levels of Cd and Zn raise significant concerns due to their potential health impacts. Cd exposure can lead to kidney dysfunction, bone fractures, and an increased risk of cancer (Li et al., 2022), while excessive Zn can cause gastrointestinal issues and disrupt the absorption of other essential minerals (Plum et al., 2010). These findings underscore the need for effective environmental monitoring and management strategies to mitigate the risks posed by Cd and Zn contamination in the region.

The PLI offers a cumulative measure of pollution by integrating the CF values of multiple metals (Jolly et al., 2023; Tomlinson et al., 1980). A PLI value greater than one signifies pollution, while values below one indicate no pollution. In our study, the PLI values across all sampling sites ranged from 1.11 to 2.17, indicating that the metals we investigated actively contribute to pollution at these locations. Similar findings were reported by Gupta et al. (2008), who observed comparable PLI values in wastewater-irrigated areas of West Bengal, India, highlighting significant pollution levels from industrial and agricultural sources. Furthermore, Islam et al. (2015) reported high PLI values in the Buriganga River in Bangladesh, attributing the contamination to untreated industrial effluents.

##### Ecological risk (ER) and potential ecological risk index (PERI)

Table 7 presents the potential ER and risk indicators. The reported ER values ranged from 0.89 to 3510, indicating a spectrum of ecological risks from low to very high as follows: Cd > Zn > Pb > Ni > Cu > Cr. As previously noted, Cd exhibited a remarkably high potential risk. In contrast, the ER values for Zn, except Sample 01, and other metals (Cr, Cu, and Ni) were below 40, indicating low ecological risk (Hossain et al., 2021).

**Table 7:** Ecological Risk Factor and Risk Index

Samples	Potential ecological risk factor						Risk index
	**Cr**	**Cd**	**Ni**	**Cu**	**Zn**	**Pb**	
Sample 01	0.89	3242.00	10.65	4.60	50.87	27.38	3336.39
Sample 02	1.60	3510.00	10.79	9.51	29.47	20.62	3581.98
Sample 03	2.24	1464.00	10.02	8.42	22.69	17.98	1525.36
Sample 04	1.44	1599.00	0.89	3.62	21.13	15.55	1641.63
Sample 05	1.56	1139.00	1.01	3.63	20.88	19.60	1185.68

Particularly when assessing ecological risks in aquatic areas, PERI is essential to environmental evaluations. It makes it easier to identify areas with higher pollution levels by systematically assessing the amounts of contamination from different metals (Tiabou et al., 2024). It must be noted that the elevated PERI reported for metals such as Cr, Cd, Ni, Cu, Zn, Pb, Fe, and Mn across all sampling stations (S-1 to S-5) was primarily driven by the elevated levels of Cd. For instance, Sample S-1 exhibited a total risk index of 3336.39, with Cd alone contributing 3242.00 (Table 7). This indicates that Cd is the dominant contaminant at this site, likely due to industrial effluents and agricultural runoff.

In contrast, the other metals, including Cr, Cu, Ni, and Zn, displayed much lower ER values, indicating a relatively lower ecological risk. For example, the ER values for Cr ranged from 0.89 to 2.24, suggesting minimal pollution. Similarly, Cu exhibited Er values from 3.62 to 9.51 and Zn from 20.88 to 50.87, which are comparatively lower than Cd.

##### Related risk assessment of human health

Analysis of soil samples from the study area revealed the non-carcinogenic HQ for adults and children across various exposure pathways for Cr, Ni, Cu, Zn, Cd, and Pb (Table 8). Ingestion presented the highest HQ values, following the sequence Pb > Cr ≥ Cd > Zn > Ni > Cu for both age groups. Inhalation and dermal contact showed different patterns, with Zn and Cd posing the highest risk, respectively. Importantly, none of the HQ values exceeded the threshold of 1 for any individual exposure route, except for Cd and Cr via dermal contact, indicating no significant non-carcinogenic health risk through ingestion or inhalation. This aligns with findings by Mizan et al. (2023), who similarly reported that Cr and Cd exceeded the HQ for dermal exposure in both adults and children, highlighting this pathway as a potential concern for these specific metals.

**Table 8:** The total carcinogenic risk (CR) and target hazard index (HI) of heavy metals through ingestion, dermal absorption, and inhalation of soil

Metals	Non-carcinogenic health risk (HQ)		Carcinogenic health risk (CR)	
	Adult	Child	Adult	Child
Ingestion				
Cr	3.0 × 10^–2^	3.0 × 10^–1^	2.73 × 10^–5^	1.76 × 10^–4^
Cd	3.0 × 10^–2^	2.8 × 10^–1^	1.32 × 10^–5^	3.53 × 10^–4^
Ni	6.2 × 10^–3^	5.8 × 10^–2^	7.31 × 10^–6^	1.95 × 10^–4^
Cu	1.8 × 10^–3^	1.7 × 10^–2^	NC	NC
Zn	1.3 × 10^–2^	1.2 × 10^–1^	NC	NC
Pb	3.2 × 10^–2^	3.0 × 10^–1^	6.59 × 10^–8^	1.76 × 10^–6^
Inhalation				
Cr	6.4 × 10^–3^	3.0 × 10^–2^	5.89 × 10^–7^	5.89 × 10^–7^
Cd	6.0 × 10^–3^	2.8 × 10^–2^	2.78 × 10^–8^	2.78 × 10^–8^
Ni	1.2 × 10^–3^	5.8 × 10^–3^	1.66 × 10^–8^	1.66 × 10^–8^
Cu	3.7 × 10^–4^	1.7 × 10^–3^	NC	NC
Zn	1.3 × 10^–2^	5.9 × 10^–2^	NC	NC
Pb	6.3 × 10^–3^	3.0 × 10^–2^	6.85 × 10^–7^	6.85 × 10^–7^
Dermal				
Cr	6.34	4.74	2.03 × 10^–8^	7.58 × 10^–8^
Cd	11.97	8.96	1.89 × 10^–8^	7.06 × 10^–8^
Ni	0.09	0.07	1.67 × 10^–8^	6.24 × 10^–8^
Cu	0.02	0.02	NC	NC
Zn	0.25	0.19	NC	NC
Pb	0.84	0.63	3.76 × 10^–9^	1.41 × 10^–8^
	Hazard Index (HI)		Total lifetime cancer risk	
	19.51	14.61	1.925 × 10^–5^	7.273 × 10^–4^

The cumulative human health risk assessment (hazard index; HI) was calculated for both adults and children, resulting in values of 19.51 and 14.61, respectively (Table 8). These values exceeding 1 indicate potential adverse health effects from combined exposure to the assessed heavy metals (Hossain et al., 2021). Given the International Agency for Research on Cancer (IARC) classification of Cr, Ni, Cd, and Pb as carcinogenic (IARC 2011), we further evaluated the cancer risk for both adults and children. This assessment is crucial for understanding the long-term health implications of exposure to these metals and informing appropriate risk management strategies (Ali et al., 2019).

The estimated carcinogenic risks for Cr, Ni, Cd, and Pb across all exposure routes generally fell within the acceptable (< 10^−6^) and tolerable (10^−6^ to 10^−4^) ranges for both adults and children (Table 8). However, the carcinogenic risk for Cr, Cd, and Ni through ingestion approached the upper limit of the tolerable range for children, suggesting a potential concern. The risk for these metals via ingestion remained tolerable for adults.

### Waste water analysis

#### Correlation analysis

**Fig. 5:**
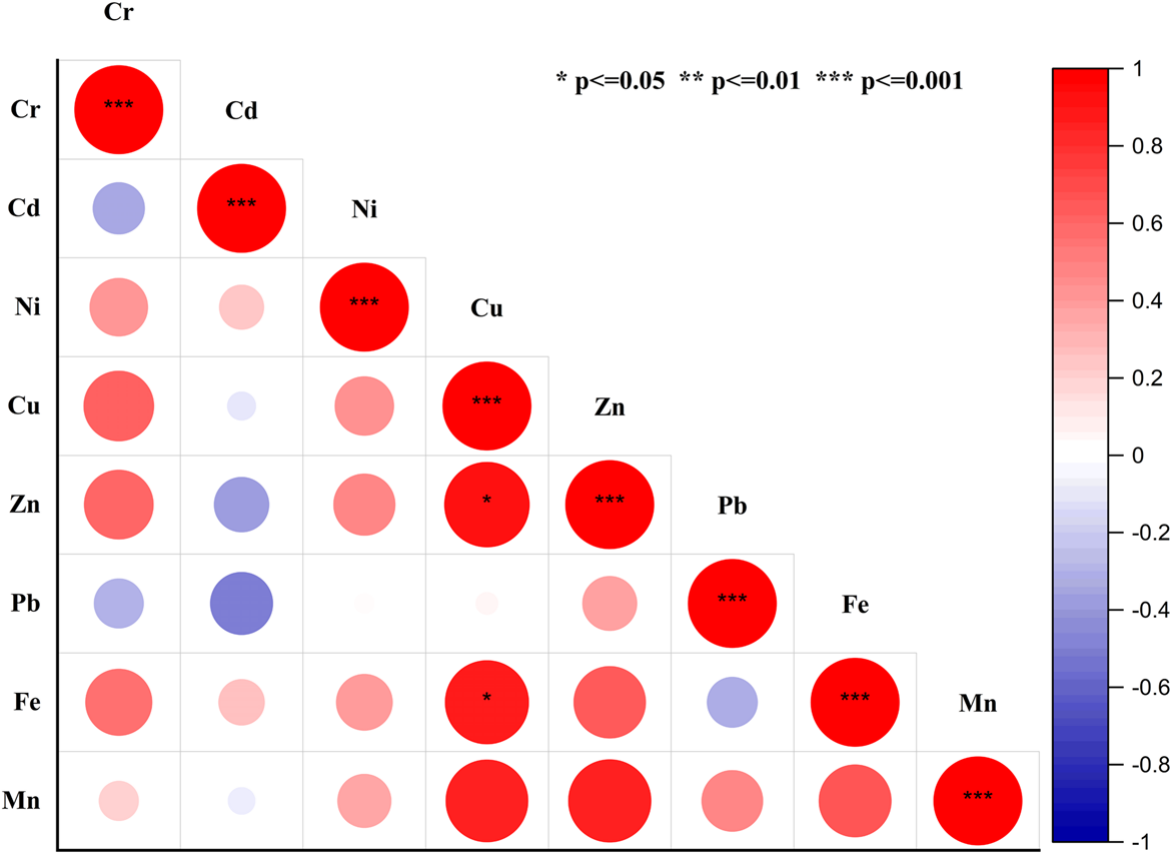
The Matrix of Pearson Correlation Coefficient for the Heavy Metals in the Wastewater sample

The correlation between the heavy metals in the wastewater samples from the study region is displayed in Fig. 5 using Pearson's correlation statistics (Hashem et al., 2021). The dataset was tested for normality prior to Pearson correlation analysis, and approximately 75% of the variables exhibited a normal distribution (data not shown), justifying the use of this method to identify relationships between the heavy metals. Notably, there were strong positive correlations between various metal pairs such as Cu–Zn (0.92), Cu-Fe (0.90), Zn-Fe (0.66), Cu-Mn (0.86), Zn-Mn (0.87), and Fe–Mn (0.67). These strong positive correlations suggest that these metals likely originated from similar industrial sources (Mansouri et al., 2012). For instance, Cu and Zn are commonly found together in effluents from metal plating and manufacturing industries and the corrosion of galvanised steel structures (Patel et al., 2018). The correlation between Cu and Fe might be attributed to metal processing industries, where both metals are used extensively (Bhuyan et al., 2017). Similarly, the strong correlations involving Mn with Cu, Zn, and Fe could be linked to industrial activities such as alloy production and steel manufacturing or mainly due to the unmanaged dumping of municipal waste close to river catchments (Borah et al., 2020). Moderate positive correlations (Fig. 5), such as Cr-Cu (0.63), Cr-Zn (0.62), and Cr-Fe (0.57), indicate some shared sources, such as electroplating industries, which often use Cr and Cu in their processes, as well as paint and pigment manufacturing where both Cr and Zn are utilised. For example, Feng and Pan (2023) identified similar sources in the Liangtan River, where industrial activities related to electroplating and metal finishing contributed to the contamination. In contrast, low positive correlations (Fig. 5), including Ni–Cr (0.43) and Ni-Cu (0.45), suggest minor co-sourcing from industrial activities such as stainless-steel production and electroplating, where nickel is used in smaller quantities alongside chromium and copper (Hama Aziz et al., 2023).

The distinct negative correlations (Fig. 5) involving Cd, such as Cd-Cr (−0.34) and Cd-Zn (−0.39), suggest that Cd may originate from different industrial sources, such as metal industries, mining activities, traffic emissions, and sewage sludge (Sidhu et al., 2017) or agricultural activities, such as the use of phosphate fertilisers, which often contain Cd as an impurity (Kubier & Pichler, 2019). We stress that understanding such correlations is crucial for identifying pollution sources as it highlights the need for urgent attention to implementing broader pollution control measures in industrial areas such as Gazipur.

#### Physiochemical parameters of the collected samples

The physicochemical characteristics of the wastewater in the research region are shown in Table 9. The pH level of a water system is crucial for its suitability for various uses. Both excessively high and low pH levels can be harmful to aquatic life (Morrison et al., 2001), as they affect the solubility of essential metals and other chemical.

**Table 9:** Physiochemical parameters of wastewater

Sample ID	Parameters				
	pH	EC (mS/cm)	DO (mg/L)	COD (mg/L)	TDS (mg/L)
Sample 01	7.621	1.75	0.41	189	602
Sample 02	7.350	2.17	0.47	293	585
Sample 03	7.421	2.14	0.34	308	510
Sample 04	7.891	2.16	0.40	524	570
Sample 05	7.591	2.19	0.35	255	630
*BECR 2023	6–9	1.2	4.5–8	125	2100

*Bangladesh standards for discharging wastewater

contaminants in the water, which can subsequently harm the environment and the people who depend on it (Odjadjare & Okoh, 2010). For example, a pH level above 9.5 can be detrimental to aquatic organisms (Hashem et al., 2021). In this study, the pH values ranged from 7.3 to 7.8 (Table 9), all within the permissible limits of the Bangladesh Environmental Conservation Rules (Bangladesh Environmental Conservation Rules 2023). Kachoueiyan et al. (2023) showed that a higher solution redox potential will enhance the release of HMs from sediments into the water, thereby lowering the pH.

These findings suggest that, based on pH criteria, the effluent is unlikely to adversely affect the suitability of the receiving watershed for residential, fishing, and recreational purposes. EC, which serves as an indirect measure of the salinity of the water and its ability to conduct electricity, also provides insight into the water quality (Morrison et al., 2001). Sample point 05 exhibited the highest EC (Table 9), indicating elevated levels of dissolved salts, particularly chlorides, which likely contributed to the increased EC at this location (Mamba et al., 2012). Except for sampling point 01, all sampling points displayed EC levels that exceeded the permissible limits of the Bangladesh Environmental Conservation Rules 2023, suggesting potential salinity issues across most of the sampled sites.

The TDS measurements in the study ranged from 510 mg/L to 630 mg/L (Table 9). These values are within the permissible limits for effluents discharged into surface waterways, which is ≤ 2,000 mg/L per WHO guidelines (Odjadjare & Okoh, 2010) and comply with permissible wastewater discharge standards in Bangladesh.

COD measures the amount of oxygen required by a strong oxidant, such as sulfuric acid (H_2_SO_4_), to decompose organic and inorganic components in a water system. High COD levels indicate severe oxygen depletion, which can negatively impact aquatic biota (Fatoki et al., 2003).

In this study, COD values ranged from 189 mg/L to 524 mg/L, exceeding the acceptable limit of 125 mg/L recommended by the Bangladeshi government for wastewater discharge into surface waters (Table 9). The elevated COD levels suggest substantial contamination from organic pollutants, likely due to industrial discharges and agricultural runoff containing high levels of organic matter (Aoki et al., 2004).

DO is essential for maintaining the oxygen balance within an aquatic ecosystem. This study revealed that the DO levels in the area are significantly below the acceptable limit set by the Bangladesh Environmental Conservation Rules 2023. Low DO levels adversely affect aquatic life by increasing susceptibility to disease, impairing swimming ability, and disrupting the feeding, migration, reproduction, and survival of fish and other aquatic organisms (Odjadjare & Okoh, 2010). The low DO levels observed in the study are likely a result of the high COD levels. The high demand for oxygen to decompose the abundant organic pollutants depletes the available dissolved oxygen in the water, creating a stressful environment for aquatic life (Aoki et al., 2004).

### Heavy metal analysis in plant samples

#### Heavy metals in grass

Table 10 presents the concentration ranges, mean values, and standard deviations of the heavy metals studied in grass samples* (E. fluctuens)*. Additionally, the measured concentrations of these metals were compared to the acceptable limits set by Bangladesh's food safety authorities (BFSA) and the FAO/WHO guidelines. Most metals in the data set exceeded the BFSA and WHO guideline limits, except for Mn.

**Table 10:** Heavy metal content in the grass

Heavy metals	Range	Mean	STDEV	FAO / WHO	BFSA 2013	Unit
Cr	1.246–27.232	15.4502	12.02487	2.30	1.0	mg/kg
Cd	4.89–15.72	11.224	4.991566	0.20	0.20	mg/kg
Ni	1.223–16.954	11.7144	6.190758	2.70	1.0	mg/kg
Cu	9.995–41.558	23.2306	12.74138	10	NA	mg/kg
Zn	1425.97–2320.75	1955.77	361.7266	50	NA	mg/kg
Pb	10.254–38.88	22.3156	12.76178	0.30	0.30	mg/kg
Fe	915.16–1994.55	1560.188	523.891	425	NA	mg/kg
Mn	49.248–172.716	96.1618	49.38541	500	NA	mg/kg

*NA* Not available; *BFSA* Bangladesh Food Safety Authority

The concentration of Zn was particularly high, ranging from 1425.97 mg/kg to 2320.75 mg/kg, with the highest concentration observed in sample 03 (Table 10). The Zn levels exceeded the FAO/WHO maximum permissible limit by more than 40 times (FAO/WHO, 2011). This elevated concentration can be attributed to the high accumulation capacity of grass and vegetables for Zn compared to other metals. Hashem et al. (2021) also noted that the rubber industry in the study region could be a significant source of Zn contamination.

Ni concentrations were the lowest among the metals studied, with only sample 04 reported below the acceptable limits. This suggests relatively low Ni deposition in the vegetables. Cr concentrations showed a similar trend, where all samples exceeded the Bangladesh Environmental Conservation Rules (2023) maximum allowable amount, except for sample 05, which was within the FAO/WHO (2011) acceptable limits. Hashem et al. (2021) suggested that the plastics recycling industry, which uses substantial amounts of Cr for plating, could release significant quantities of Cr into the environment, leading to its high accumulation in local vegetation. The Pb concentrations in the vegetable samples ranged from 10.254 mg/kg to 38.88 mg/kg (Table 10), exceeding the safe limits set by both the Bangladesh Food Safety Authority (2013) and FAO/WHO (2011). Similar Pb concentrations (17.00–25.00 mg/kg) were reported by Sharma et al. (2006) in vegetables grown in industrial soils. Pb contamination is known to impede plant growth, disrupt photosynthesis, darken roots, interfere with mineral nutrition, affect water balance, alter hormone levels, degrade membrane structures, and cause adverse effects (Ali & Nas, 2018).

The detected Cd content in the studied region exceeded the limit set by FAO/WHO (2011) and BFSA (2013) by almost 55 times (0.20 mg/kg; Table 10). Once absorbed by the roots, Cd is readily transported throughout the plant, with concentrations in the roots typically being at least double those in the vegetative tops (Koeppe, 1977). This high level of Cd poses significant health risks, as Cd is a known carcinogen and can cause kidney damage, bone fractures, and other serious health issues.

Cu is essential for plant growth and development but becomes toxic at high concentrations, adversely affecting photosynthesis and plant metabolism (Lin & Jin, 2018). In this study, Cu concentrations, except for sample 05, were close to or exceeded the FAO/WHO maximum allowable limit (2011), indicating potential toxicity risks to plants and, subsequently, to humans consuming these contaminated vegetables. Fe is required in trace amounts for plant growth; however, excessive Fe can be highly toxic (Manzoor et al., 2018). All the samples (Table 10) in this study surpassed the standard limit of 425 mg/kg set by FAO/WHO (2011). Excessive Fe can lead to oxidative stress in plants, causing damage to cellular structures and impairing growth. High levels of Fe in the food chain can also pose health risks to humans, including gastrointestinal issues and potential long-term effects on organs.

#### Soil-grass transfer coefficient (TF)

The TF, also known as the plant concentration factor (PCF), is a crucial metric used to quantify the movement of trace hazardous chemicals from the soil into plant tissues (Ali et al., 2022). This metric is defined as the ratio of the concentration of a metal in the plant to its concentration in the soil. The TF or PCF is fundamental in assessing the risk of human exposure to metals through the food chain, particularly from crops irrigated with wastewater (Cui et al., 2004). Evaluating the TF is essential for predicting the bioaccumulation of metals in plants from soils, thereby helping to gauge the potential health risks associated with contaminated soils (Kachenko et al. 2006; Ali et al., 2022).

Table 11 presents the TF values, which reflect the bioavailability of metals in the plants under study. The observed TF pattern for trace metals in the vegetable samples was as follows: Mn ≥ Ni > Zn ≥ Cd > Fe > Cu > Cr > Pb. This pattern indicates that Mn is relatively weakly retained by the soil and is more readily taken up by plants, likely due to its high mobility and essential role in plant physiology, which facilitates its absorption and translocation within plant tissues (Alam et al., 2020).

**Table 11:** Soil-vegetable transfer coefficient (TF)

Samples	Cr	Cd	Ni	Cu	Zn	Pb	Fe	Mn
01	0.549	0.443	0.093	0.556	0.295	0.355	0.897	0.228
02	0.052	0.193	0.079	0.150	0.800	0.220	0.495	0.316
03	0.270	1.074	0.124	0.548	1.077	0.452	0.924	1.107
04	0.356	0.898	0.101	0.884	0.893	0.165	0.911	0.877
05	0.018	0.429	1.106	0.306	1.007	0.150	0.430	0.516
Mean	0.249	0.608	0.301	0.489	0.815	0.268	0.732	0.609
Std. Dev	0.220	0.365	0.450	0.279	0.309	0.130	0.247	0.374
Min	0.018	0.193	0.079	0.150	0.295	0.150	0.430	0.228
Max	0.549	1.074	1.106	0.884	1.077	0.452	0.924	1.107

In contrast, Pb exhibits substantial sorption (Table 11) to soil colloids, reducing its bioavailability to plants. This strong sorption can be attributed to its tendency to form stable complexes with organic matter and clay minerals in the soil, which limits its mobility and uptake by plants (Xu et al., 2022). Additionally, the chemical form of Pb in the soil, which often precipitates as insoluble salts, further decreases its bioavailability (Ruby et al., 1996) (Table 8).

## Conclusion

The emerging crisis of metal contamination in our water, soil, and food chain poses a serious threat to both environmental and human health. This is mainly driven by rapid urbanisation, industrial expansion, and the lack of proper regulations in many countries worldwide. As these metals permeate the environment and bioaccumulate, they trigger a cascade of adverse effects, ranging from acute toxicity to chronic diseases, disrupting ecosystems and undermining agricultural productivity.

Our comprehensive investigation reveals a considerable issue of heavy metal contamination in the Gazipur region. Cd and Zn are identified as primary contaminants from anthropogenic sources such as industrial effluents and agricultural runoff. The elevated contamination levels, supported by various indices (EF, I_geo_, CF, PERI), pose substantial ecological risks, particularly with Cd exhibiting the highest potential hazard. The physicochemical analysis of wastewater further emphasises the deteriorating water quality, with high salinity and organic pollution levels further threatening aquatic ecosystems.

In addition, the human health risk assessment underscores the potential adverse health effects, especially for children, due to dermal exposure to Pb, Cr, and Cd. The elevated hazard indices and lifetime cancer risk estimates, particularly for Cr, Cd, and Ni through ingestion, highlight the urgent need for effective risk management strategies. The bioavailability and transfer of metals from soil to vegetables, especially Mn, further underscore the potential for human exposure through the food chain.

In conclusion, our findings provide compelling evidence for immediate and comprehensive intervention to mitigate heavy metal pollution in the Gazipur region. Implementing stringent industrial effluent treatment, promoting sustainable agricultural practices, and ensuring safe waste disposal are imperative steps towards protecting public health and the environment. Furthermore, continuous monitoring and research efforts are essential to tracking contamination levels, assessing long-term health impacts, and refining mitigation strategies for the future.

## Data Availability

All data on the heavy metal analysis of water, soil and plant material used for the indicating of ecological risks that support the findings of this study are included within this paper.
